# Speeding Up and Improving Image Quality in Glioblastoma MRI Protocol by Deep Learning Image Reconstruction

**DOI:** 10.3390/cancers16101827

**Published:** 2024-05-10

**Authors:** Georg Gohla, Till-Karsten Hauser, Paula Bombach, Daniel Feucht, Arne Estler, Antje Bornemann, Leonie Zerweck, Eliane Weinbrenner, Ulrike Ernemann, Christer Ruff

**Affiliations:** 1 Department of Diagnostic and Interventional Neuroradiology, Eberhard Karls-University Tübingen, 72076 Tübingen, Germany; till-karsten.hauser@med.uni-tuebingen.de (T.-K.H.); arne.estler@med.uni-tuebingen.de (A.E.); leonie.zerweck@med.uni-tuebingen.de (L.Z.); eliane.weinbrenner@med.uni-tuebingen.de (E.W.); ulrike.ernemann@med.uni-tuebingen.de (U.E.); christer.ruff@med.uni-tuebingen.de (C.R.); 2 Department of Neurology and Interdisciplinary Neuro-Oncology, University Hospital Tübingen, Hoppe-Seyler-Str. 3, 72076 Tübingen, Germany; paula.bombach@med.uni-tuebingen.de; 3 Hertie Institute for Clinical Brain Research, Eberhard Karls University Tübingen Center of Neuro-Oncology, Ottfried-Müller-Straße 27, 72076 Tübingen, Germany; 4 Center for Neuro-Oncology, Comprehensive Cancer Center Tübingen-Stuttgart, University Hospital of Tuebingen, Eberhard Karls University of Tübingen, Herrenberger Straße 23, 72070 Tübingen, Germany; 5 Department of Neurosurgery, University Hospital Tübingen, Hoppe-Seyler-Str. 3, 72076 Tübingen, Germany; daniel.feucht@med.uni-tuebingen.de; 6 Department of Neuropathology, Institute of Pathology and Neuropathology, University Hospital Tübingen, Calwerstraße 3, 72076 Tübingen, Germany; antje.bornemann@med.uni-tuebingen.de

**Keywords:** glioblastoma, MRI, deep learning-based reconstruction, acceleration of acquisition time, image quality assessment, diagnostic accuracy, RANO 2.0

## Abstract

**Simple Summary:**

Interest in applying artificial intelligence to medical imaging to enhance image quality has grown in both clinical practice and research. Nonetheless, these artificial intelligence models require further validation for use in diagnosing and monitoring disease progression to effectively compare them with traditional techniques. This study aimed to assess the effectiveness of a deep learning-optimized glioma protocol for MRI scans in patients with glioblastoma. The study protocol focuses on improving image quality and shortening scan time, which might be particularly beneficial for critically ill or uncooperative patients. The results showed that deep learning-reconstructed sequences can save scan time and simultaneously increase subjective image quality but with less subjective image noise. In addition, tumor volume measurements and neuro-oncologic response assessments were not affected negatively by deep learning. This study demonstrated the feasibility of the deep learning protocol for MRI of glioblastomas.

**Abstract:**

A fully diagnostic MRI glioma protocol is key to monitoring therapy assessment but is time-consuming and especially challenging in critically ill and uncooperative patients. Artificial intelligence demonstrated promise in reducing scan time and improving image quality simultaneously. The purpose of this study was to investigate the diagnostic performance, the impact on acquisition acceleration, and the image quality of a deep learning optimized glioma protocol of the brain. Thirty-three patients with histologically confirmed glioblastoma underwent standardized brain tumor imaging according to the glioma consensus recommendations on a 3-Tesla MRI scanner. Conventional and deep learning-reconstructed (DLR) fluid-attenuated inversion recovery, and T2- and T1-weighted contrast-enhanced Turbo spin echo images with an improved in-plane resolution, i.e., super-resolution, were acquired. Two experienced neuroradiologists independently evaluated the image datasets for subjective image quality, diagnostic confidence, tumor conspicuity, noise levels, artifacts, and sharpness. In addition, the tumor volume was measured in the image datasets according to Response Assessment in Neuro-Oncology (RANO) 2.0, as well as compared between both imaging techniques, and various clinical–pathological parameters were determined. The average time saving of DLR sequences was 30% per MRI sequence. Simultaneously, DLR sequences showed superior overall image quality (all *p* < 0.001), improved tumor conspicuity and image sharpness (all *p* < 0.001, respectively), and less image noise (all *p* < 0.001), while maintaining diagnostic confidence (all *p* > 0.05), compared to conventional images. Regarding RANO 2.0, the volume of non-enhancing non-target lesions (*p* = 0.963), enhancing target lesions (*p* = 0.993), and enhancing non-target lesions (*p* = 0.951) did not differ between reconstruction types. The feasibility of the deep learning-optimized glioma protocol was demonstrated with a 30% reduction in acquisition time on average and an increased in-plane resolution. The evaluated DLR sequences improved subjective image quality and maintained diagnostic accuracy in tumor detection and tumor classification according to RANO 2.0.

## 1. Introduction

Glioblastomas represent 12–15% of all brain tumors [1]. With an incidence rate of 3.21 per 100,000 population, they stand out as the most prevalent and most aggressive malignant brain tumors in adults [2]. The diagnostic and monitoring processes for patients with glioblastomas are based on MRI examinations. These imaging studies have time-consuming protocols and are pivotal for classifying tumor type, determining tumor grade, and monitoring tumor response to treatment, as well as detecting potential tumor recurrence.

However, challenges can arise, especially in patients with tumor-related reduced patient compliance. In these cases, lengthy examination protocols can lead to a decreased MRI image quality, an increase in motion artifacts, or even premature termination of the examination. Traditional methods to accelerate the examination time, such as parallel acquisition techniques (PATs) and compressed sensing (CS), have been employed to mitigate these challenges [3,4]. However, PAT can result in a reduction of signal-to-noise ratio proportional to the square-root of the PAT factor, while CS may yield unrealistically smooth images [5,6,7].

In response to these challenges, the use of artificial intelligence (AI) in medical imaging has received significant attention and revolutionized the field [8]. AI has brought about notable enhancements in image quality and the integration of neural network functions [9,10]. Within this context, deep learning (DL)-based methods have emerged as a promising approach to address the limitations of conventional acceleration techniques and to simultaneously increase efficiency, image quality, and precision, while reducing the scan time [11,12].

Numerous studies have underscored the efficacy of AI-based methods in enhancing the quality of neuroradiological state-of-the-art fluid-attenuated inversion recovery (FLAIR) sequences and T1-weighted and T2-weighted images, as well as reducing the acquisition time across various imaging scenarios [13,14,15,16].

Integrating DL reconstruction (DLR) techniques into medical imaging offers the potential to improve diagnostic accuracy and efficiency in glioma patients. Nevertheless, further research is warranted to systematically investigate the quality of DL-generated images and optimize their practical application in routine clinical settings.

This clinical study aimed to investigate the technical feasibility, image quality, and diagnostic accuracy of a DL-based glioma protocol consisting of novel T1-weighted and T2-weighted turbo spin echo (TSE) sequences and FLAIR imaging in patients with glioblastoma. The assessment addresses the acquisition time, subjective image quality, robustness to artifacts, and diagnostic confidence. The research results may offer valuable insights into the feasibility and practicality of implementing DLR methods in clinical glioma imaging.

## 2. Materials and Methods

### 2.1. Study Design

**Figure 1:**
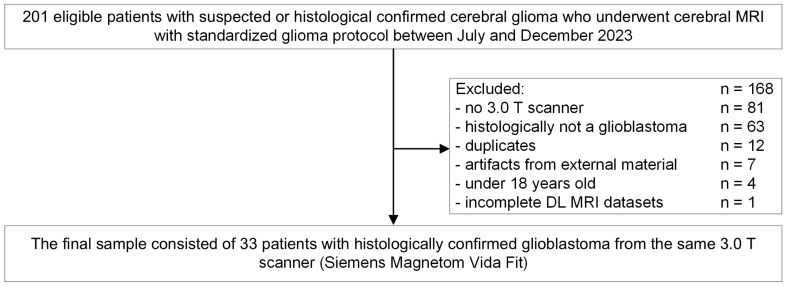
Study flowchart and patient enrollment. T = Tesla. DL = deep learning.

This monocentric retrospective study was approved by the institutional review board under the code 761/2023B02. All study procedures were in line with the declaration of Helsinki and its later amendments. The study includes 33 glioblastoma patients who had undergone cerebral MRI for therapy assessment or first diagnosis between July 2023 and December 2023. Exclusion criteria were non-conditional MRI implants, duplicates, artifacts from external material, being under the age of 18, and having incomplete DL-based MRI datasets (Figure 1).

### 2.2. Imaging Protocol and Deep Learning Reconstruction Algorithm

All examinations were performed using a 3-Tesla clinical MRI scanner (MAGNETOM Vida Fit, Siemens Healthineers, Erlangen, Germany). Patients were scanned in a supine position, using a setup of a 32-channel head coil. The institution’s standard glioma protocol according to the consensus recommendations for standardized brain tumor imaging [17] consists of the following sequences: FLAIR in axial plane, T1-weighted pre- and postcontrast TSE imaging in axial plane (slice thickness: 4 mm), T1-weighted three-dimension magnetization-prepared rapid gradient-echo pre- and postcontrast imaging in sagittal plane, and T2-weighted TSE imaging in two planes (axial with slice thickness, 4 mm; coronal with slice thickness, 3 mm); diffusion-weighted imaging with two different acquired b-values in axial plane (0 s/mm^2^, 1000 s/mm^2^); and apparent diffusion coefficient mapping and perfusion imaging, using a flow rate of 3 mL/s followed by a saline flush of 20 mL (0.1 mmol/kg body weight gadobutrol; Gadovist, Bayer Healthcare, Leverkusen, Germany), as well as accelerated, DLR sequences for FLAIR, contrast-enhanced T1-weighted, and T2-weighted TSE imaging with the same planes and slice thickness. For standard axial FLAIR (FLAIR_S_) and axial contrast-enhanced T1-weighted (T1CE_S_) and axial T2-weighted imaging (T2_S_), the acceleration factor was set to two phase-encoding (PE) steps, while for DLR axial FLAIR (FLAIR_DLR_), axial contrast-enhanced T1-weighted (T1CE_DLR_), and axial T2-weighted imaging (T2_DLR_), it was set to four PE steps. Reconstruction of images can be accomplished through the utilization of the existing standard clinical hardware infrastructure of the MRI scanner workstation without any clinically noticeable time delay. In-plane resolution was improved and typically doubled in accelerated sequences compared to standard state-of-the-art TSE sequences with simultaneously reduced acquisition times. Detailed acquisition parameters for these sequences and their DLR versions with improved in-plane resolution are displayed in Table 1.

The study utilized an unrolled variational network, which was previously recognized for its potential to accelerate acquisition time across different applications, for DLR. The utilized algorithm facilitates efficacious denoising, thereby enhancing the visual signal-to-noise ratio of the images. The network underwent training and certification processes, using over 10,000 slices obtained from volunteer acquisitions conducted on various clinical 1.5-Tesla and 3-Tesla scanners (MAGNETOM scanners, Siemens Healthcare, Erlangen, Germany). Following training, the certified network was integrated into the scanner’s reconstruction pipeline by the vendor.

The image reconstruction algorithm employed in this study follows the methodology outlined by Herrmann et al. [18]. The image reconstruction offers the option of either a fixed iterative reconstruction method or a variational network approach [19,20]. In this research, the deep neural network model integrates both physical principles and data-driven approaches for MRI. The model utilizes a fixed unrolled algorithm featuring multiple cascades that incorporate data consistency and convolutional neural network (CNN)-based regularization. The regularization model is structured hierarchically with an iterative network, which iteratively enhances memory efficiency by adjusting the resolution of feature maps. The CNN module, known as the “Deep, Iterative, Hierarchical Network”, extends the Down–Up network design through a hierarchical block arrangement. The algorithm accepts undersampled k-space data and coil sensitivity maps as input, while a separate acquisition is utilized to extract a bias field for image homogenization.

### 2.3. Image Analysis

Image analyses were conducted by two experienced neuroradiologists with eight (rater 1) and three years (rater 2) of board certification. Both raters independently evaluated 33 image datasets comprising standard and DLR FLAIR, and contrast-enhanced T1-weighted TSE and T2-weighted TSE images. All sequences were acquired in axial orientation. Furthermore, both raters were unaware of the reconstruction type, description of sequence names, patient data, and clinical and radiologic reports. The raters assessed the standard and accelerated datasets in separate sessions, randomized and mixed, with a minimum 2-week interval between sessions on a dedicated workstation with a certified image viewer software (GE Centricity PACS RA 1000 version 7.0.2; General Electric (GE) Healthcare, Chicago, IL, USA) on certified diagnostic radiology monitors (RadiForce RX350, Eizo Corporation, Hakusan, Ishikawa, Japan).

The evaluations were conducted by utilizing a Likert scale ranging from 1 to 5, with 5 representing the highest rating. Parameters assessed included image quality, diagnostic confidence, tumor conspicuity, noise levels, artifacts (encompassing pulsation artifacts and those attributed to DLR), and image sharpness. A score of 1 for image quality denoted non-diagnostic images, while a score of 5 indicated excellent quality. Diagnostic confidence was rated from 1 to 5, with 1 signifying non-diagnostic images and 5 reflecting excellent confidence. Tumor conspicuity scores ranged from 1 for very poor to 5 for excellent conspicuity. Noise levels were graded from 1 for images severely impeded by noise to 5 for noise-free images. Artifacts were assessed from 1 for excessive, highly pronounced artifacts distorting images to 5 for no artifacts present. Lastly, image sharpness was rated from 1 for severely blurred edges and non-diagnostic images to 5 for impeccable sharpness without blurring.

In a second session, both raters evaluated both the DLR and conventional datasets in an unblinded manner to compare the new DLR sequences for the presence or absence of artificial incidental findings. The conventional sequences served as ground truth.

### 2.4. RANO 2.0

The Response Assessment in Neuro-Oncology (RANO) Working Group published revised response criteria for gliomas in 2023 [21]. These RANO 2.0 criteria recommend a standard set of criteria for both high- and low-grade gliomas to be used for all trials regardless of the treatment modalities being evaluated. Response criteria for contrast-enhancing tumors, non-contrast-enhancing tumors, and tumors with both enhancing and non-enhancing components are proposed. These criteria aim to improve the assessment of response and progression in glial tumors. In this study, one board-certified neuroradiologist with 9 years of experience responsible for multicenter and therapy trials at this center performed lesions size measurements of the 33 image datasets regarding RANO 2.0 for both standard and DLR sequences, using dedicated software (mint Lesion version 3.9.2, Mint Medical GmbH, Heidelberg, Germany). The rater was blinded to the type of reconstruction. The RANO 2.0 evaluation included the detection of non-enhancing non-target lesions (NENT) by using the FLAIR sequences, the detection of enhancing target lesions (ET) with a size of at least 1.0 × 1.0 cm by using contrast-enhanced axial T1-weighted TSE images, and the detection of enhancing non-target lesions (ENT) < 1.0 cm by using contrast-enhanced axial T1-weighted TSE images. Both the area and the volume of lesions were determined for the study on conventional and DL images. On the slice of the maximum extension of the tumor, the long and short axes were indicated for the evaluation of a target or non-target lesion. The volume of the tumor lesions is decisive for the tumor response assessment according to the RANO 2.0 criteria. Based on the measurements, a consensus reading was performed by two board-certified neuroradiologists with 8 and 9 years of experience for the final RANO classification.

### 2.5. Statistical Analysis

Statistical evaluation was performed using commercially available statistical software (SPSS Statistics Version 26; IBM, Armonk, NY, USA). Continuous variables are shown using mean ± standard deviation (SD), and ordinal scaled variables are shown using median and interquartile range (IQR). Regarding the image quality-based analysis, intergroup comparisons were performed using the Wilcoxon signed-rank test. Interrater reliability and concordance were assessed using Cohen’s kappa and Kendall’s Tau. Cohen’s kappa coefficient d effect sizes were calculated and interpreted according to the following criteria: non to slight |d| = 0.01–0.2; fair |d| = 0.21–0.40; moderate |d| = 0.41–0.60; substantially large |d| = 0.41–0.60; and almost perfect agreement |d| = 0.81–1.00 [22]. Kendall’s Tau is calculated based on the difference between the number of concordant and discordant pairs in the dataset, providing a coefficient value between −1 and 1, where 0 indicates no relationship, 1 indicates a perfect agreement, and −1 indicates perfect disagreement. Kendall’s Tau effect sizes and concordance were interpreted according to the following criteria: non = 0 < 0.10; fair = 0.10 < 0.30; moderate = 0.30 < 0.50; large = 0.50 < 0.70; substantially large to almost perfect agreement = 0.70 < 1.00. The significance level alpha was set at 0.05.

## 3. Results

### 3.1. Patient Characteristics

This retrospective study included 33 patients who underwent MRI with suspected or known glioblastoma. The patients’ mean age was 59.8 ± 10.6 years, with 19 male and 14 female patients aged between 36 and 77. All tumors were histopathologically confirmed with O6-Methylguanine-DNA-methyltransferase (MGMT) promotor methylation in 17 cases (51.2%). In 2/33 patients (6%), glioblastoma was the initial diagnosis. At the time of the study imaging, one of these two patients had already recently undergone an external biopsy and received the MRI for further treatment planning; the other received a biopsy afterward. In 31/33 patients (94%), the imaging was a follow-up examination, and the time of imaging since the initial diagnosis was 23 ± 27.2 months. In total, 21/33 patients (64%) suffered from structural epilepsy. Multiple clinical tests were available at the time of study imaging. The patient’s characteristics are given in Table 2.

### 3.2. Image Quality-Based Analysis

DLR sequences shorten the acquisition time by an average of 30% compared to conventional sequences (Table 3). Remarkably, the acquisition time for the FLAIR_S_ was 2:40 min, while that for the FLAIR_DLR_ was 1:57 min. For T1-weighted TSE imaging, the T1CE_S_ acquisition time was 2:03 min, while for the T1CE_DLR_ sequence was 1:19 min. For T2-weighted TSE imaging, the T2_S_ acquisition time was 1:09 min while T2_DLR_ required 0:51 min. The added time savings for DLR imaging despite the higher in-plane resolution amounted to 26–36% compared to standard imaging.

The results of the more experienced rater 1 are described in the following. All results of both raters are available in the Table 4, Table 5 and Table 6.

**Figure 2:**
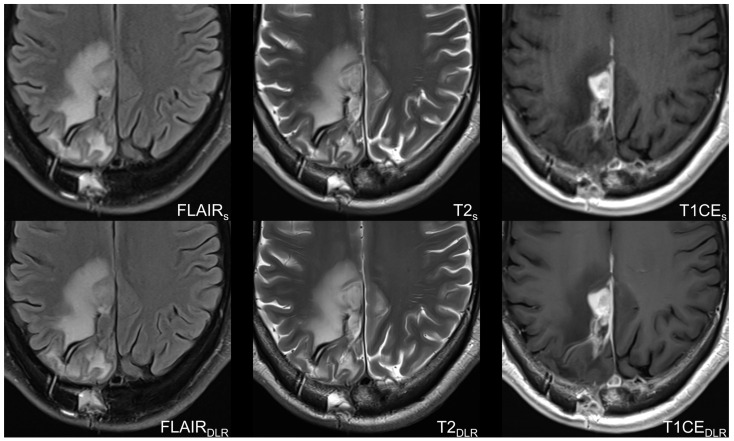
Sixty-year-old man with histologically confirmed glioblastoma in the right parietal lobe with O6-Methylguanine-DNA-methyltransferase (MGMT) promoter methylation after resection and chemoradiotherapy. Follow-up MRI 10 months after initial diagnosis showed progression of disease according to RANO 2.0. Accelerated fluid-attenuated inversion recovery images, T2-weighted images, and contrast-enhanced T1-weighted images with deep learning reconstruction (DLR) showed superior image quality, image sharpness, and tumor conspicuity compared to standard imaging technique. Furthermore, DLR images demonstrate less image noise and, especially in contrast-enhanced T1-weighted images, less motion artifacts. S = standard acquisition technique, DLR = deep learning-reconstructed technique, FLAIR = fluid-attenuated inversion recovery (images), CE = contrast-enhanced.

**Figure 3:**
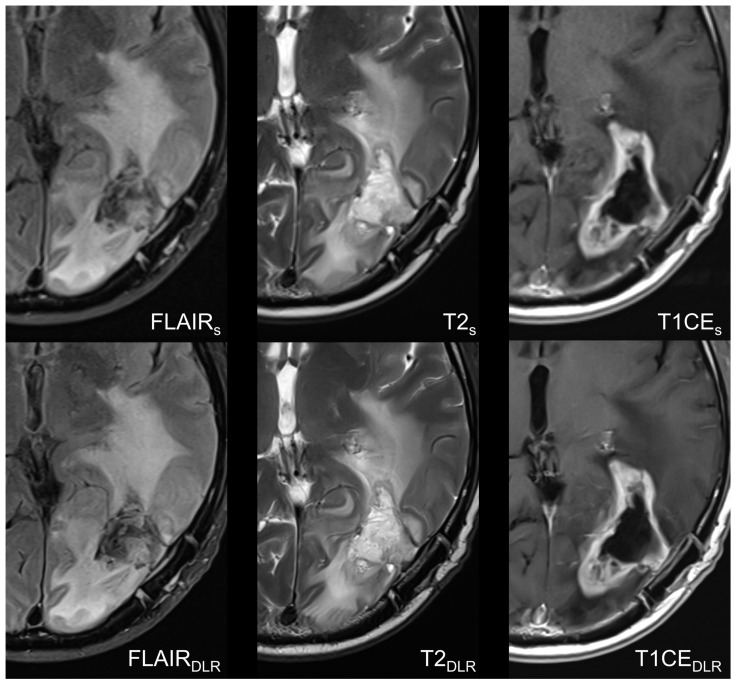
Follow-up MRI one month after radiation of a left temporo-occipital histologically confirmed glioblastoma without O6-Methylguanine-DNA-methyltransferase (MGMT) promoter methylation in a sixty-seven-year-old man. Accelerated fluid-attenuated inversion recovery images, T2-weighted images, and contrast-enhanced T1-weighted images with deep learning reconstruction (DLR) showed improved image quality, image sharpness, and lesion conspicuity compared to standard imaging technique. Consider the sharper and better delineation of the structures in and around the resection cavity in the DLR T2-weighted images compared to the standard images. In contrast, it should be noted that DLR images carry the risk of intensifying existing artifacts, as shown in this case, with the pulsation of the superior sagittal sinus. S = standard acquisition technique, DLR = deep learning-reconstructed technique, FLAIR = fluid-attenuated inversion recovery images, CE = contrast-enhanced.

Examples of imaging examinations are displayed in Figure 2 and Figure 3.

The impact and extent of image noise were rated significantly less in DLR imaging than in standard imaging (all *p* < 0.001). In FLAIR imaging, the median was 4 [IQR 4–4] for FLAIR_S_ vs. 5 [IQR 5–5] for FLAIR_DLR_ (*p* < 0.001). In T2_S_, the median was 4 [IQR 4–4] vs. 5 [IQR 5–5] for T2_DLR_ (*p* < 0.001), and for T1CE_S_, the median was 4 [IQR 4–4] vs. 5 [IQR 5–5] for T1CE_DLR_ (*p* < 0.001).

The image sharpness and overall image quality were also rated significantly better in DLR imaging than in standard imaging in all sequences studied (all *p* < 0.001). The image sharpness was rated 4 [IQR 3–4] for FLAIR_S_ vs. 5 [IQR 5–5] for FLAIR_DLR_ (*p* < 0.001), 4 [IQR 4–4] for T2_S_ vs. 5 [IQR 5–5] for T2_DLR_ (*p* < 0.001), and 3 [IQR 3–4] for T1CE_S_ vs. 5 [IQR 5–5] for T1CE_DLR_ (*p* < 0.001). The overall image quality was rated 4 [IQR 4–4] for FLAIR_S_ vs. 5 [IQR 5–5] for FLAIR_DLR_ (*p* < 0.001), 4 [IQR 4–4] for T2_S_ vs. 5 [IQR 5–5] for T2_DLR_ (*p* < 0.001), and 4 [IQR 4–4] for T1CE_S_ vs. 5 [IQR 5–5] for T1CE_DLR_ (*p* < 0.001).

Tumor conspicuity (delimitability or boundary of the tumor) was rated superior in DLR-sequences (all *p* < 0.001). FLAIR_S_ was rated with a median of 4 [IQR 4–4] vs. 5 [IQR 5–5] for FLAIR_DLR_ (*p* < 0.001), T2_S_ with a median of 4 [IQR 4–4] vs. 5 [IQR 5–5] for T2_DLR_ (*p* < 0.001), and T1CE_S_ with a median of 4 [IQR 4–4] vs. 5 [IQR 5–5] for T1CE_DLR_ (*p* < 0.001).

In terms of diagnostic confidence, no significant difference was observed between standard and DLR imaging. The median diagnostic confidence of FLAIR_S_ was 5 [IQR 5–5] vs. 5 [IQR 5–5] for FLAIR_DLR_ (*p* > 0.317). For T2_S_, the diagnostic confidence was rated with a median of 5 [IQR 5–5] vs. 5 [IQR 5–5] for T2_DLR_ (*p* = 0.157), and T1CE_S_ with a median of 5 [IQR 4–5] vs. 5 [IQR 4.5–5] for T1CE_DLR_ (*p* > 0.414).

In the unblinded, direct image comparison for the investigation of artificial incidental findings, no masked or artificial lesions were found using the DLR algorithm by either of the two board-certified neuroradiologists.

**Figure 4:**
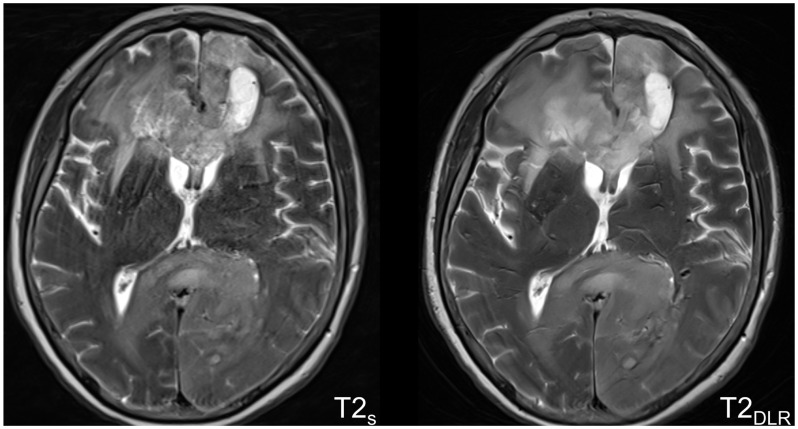
MRI of a glioblastoma of the corpus callosum in a sixty-one-year-old woman with disorientation and poor physical condition, resulting in motion-related images artifacts. Because of the faster acquisition time with the accelerated deep learning-reconstructed T2-weighted images on the right side showed less artifacts with improved overall subjective image quality, tumor conspicuity, and diagnostic confidence compared to the standard imaging technique on the left side. S = standard acquisition technique, DLR = deep learning-reconstructed technique.

In summary, we found no evidence of a difference between standard and DLR sequences regarding artifacts (>0.05), except for rater 1 regarding T1CE imaging in favor of the DLR sequence (*p* = 0.046). The most common artifacts were motion artifacts, including pulsation and banding artifacts (Figure 4).

### 3.3. Agreement and Concordance

The interrater intraprotocol agreement (standard vs. standard) for overall image quality was large to substantially large, with Kendall’s Tau values ranging from 0.571 to 0.821. The interrater intraprotocol agreement (DLR vs. DLR) was also large to substantially large, with Kendall’s Tau values ranging from 0.559 to 0.802. Similar results are shown for the interrater intraprotocol agreement for diagnostic confidence both for the standard (ranging from 0.570 to 0.784) and the DLR protocol (ranging from 0.459 to 0.803). Table 7 delineates the agreement and concordance results for Cohen’s kappa, as well as Kendall’s Tau.

### 3.4. RANO 2.0

The consensus reading by both neuroradiologists revealed no differences in the final RANO 2.0 interpretation between the standard and DLR sequences. Additionally, there were no variations in classification for small lesions in the differentiation of ET vs. ENT. The lesion size measurements between standard and DLR sequences showed no significant difference and were as follows: The volume of NENT (n = 33) was 67.76 ± 72.78 cm^3^ for FLAIR_S_ compared to 69.62 ± 72.81 cm^3^ for FLAIR_DLR_ (*p* = 0.963). The volume of ET lesions (n = 12) was 45.92 ± 52.58 cm^3^ for T1CE_S_ compared to 45.69 ± 52.75 cm^3^ for T1CE_DLR_ (*p* = 0.993) and of ENT lesions (n = 6) 0.48 ± 0.38 cm^3^ for T1CE_S_ compared to 0.49 ± 0.37 cm^3^ for T1CE_DLR_ (*p* = 0.951).

## 4. Discussion

The use of DL for MRI reconstructions has the potential to reduce the examination time for TSE and FLAIR acquisitions. In recent years, a number of different approaches to deep learning (DL) have emerged, and a growing number of new developments in artificial networks have initiated a paradigm shift in the field of medical imaging [23,24,25,26].

However, the literature is missing studies applying DLR to rapidly and undersampled acquired MRI datasets of glioma protocols in real clinical contexts. Our research investigated whether DLR significantly reduces MRI scan times by an average of 30% per sequence and improves image quality simultaneously compared to conventional TSE and FLAIR sequences.

These results are consistent with the literature, which showed a scan-time reduction for neuroradiological and non-neuroradiological applications [27,28,29,30,31,32]. Obtaining a consistently high image quality during the daily care of elderly, ill, and uncooperative patients can be a complex task. To solve this problem, several new techniques have been introduced in recent decades. Before the advent of DLR, high levels of acceleration exceeding the Nyquist–Shannon sampling limit were achievable through CS [33]. In contrast to traditional acceleration methods like CS, DL-based acceleration maintains image quality and resolution through the integration of physical modeling via coil sensitivity into the adaptable neural network framework [20,34,35]. The integration of DLR enables a greater degree of subsampling compared to post-processing methodologies, resulting in faster image acquisition [36,37]. Significantly accelerating and reducing examination times could represent a crucial component in improving healthcare delivery and addressing the balance between supply and demand. Uncooperative or restless patients, such as those with late-stage glioblastoma, generally require more time for an MRI examination than healthy subjects of the same age. Regarding movement artifacts, there is a risk that diagnostically unusable sequences are recorded, which have to be repeated after the patient has been well encouraged. In the worst case, this can even lead to the examination being aborted. The new technology offers a clear advantage, especially for patients who can only lie still for a certain period of time and only become restless after a certain time. DLR sequences allow for more diagnostically usable sequences to be acquired in the same amount of time, so that the patient does not even reach the point of onset of agitation. However, it is important to note that there are several components of an MRI examination that cannot be abbreviated, and the reduction in the acquisition time of sequences, therefore, does not fully correspond to the reduction in the total undercutting time. These factors include informed consent and patient preparation (e.g., intravenous access), patient positioning, localization at the start of the examination, sequence planning, and contrast administration. Balancing optimal medical care with economic efficiency has long been a challenging task in medicine. Healthcare systems around the world continue to be challenged by financial constraints that contrast with the rising demand driven by costly therapies, increased life expectancy, and advances in diagnostic technology. By reducing the acquisition time of the FLAIR and TSE sequences by 30%, a maximum overall time savings of 20–25% can be expected for the entire MRI examination. This reduction in time would result in a corresponding reduction in cost. However, since the financial reimbursement and the costs for equipment, electricity and personnel vary greatly from country to country, no general and useful statement can be made here about absolute costs. Furthermore, minimizing examination time enables higher patient comfort, and a greater throughput of patients undergoing examinations, yielding not only economic benefits but also meeting the demands of a high volume of examination requests.

Another advantage of DLR is the improved image quality despite the reduced acquisition time. This is an important finding, as inconsistencies (“instabilities”) can occur during DLR. These inconsistencies can manifest themselves in the suppression of small pathological features (“masking”) or in the creation of artificial elements (“artifacts”) in the image [38]. We showed that no additional artificial findings or masking of relevant findings was generated in our used DLR images. This result is consistent with previous research on the mechanisms of DLR, where comparable findings were obtained [14,15,39]. Another type of artifact, banding artifacts, is known to be a by-product of Cartesian DLR, especially in image areas with low signal-to-noise ratios. These artifacts appear as streaks aligned with the direction of phase encoding [14]. However, within the study population, no statistically significant difference in the presence of artifacts was found between the two compared reconstruction protocols. These observations are in line with previous findings in the evaluation of DLR spine images [14].

The subjective image-quality assessment indicated an increased overall image quality, tumor conspicuity, image sharpness, and less image noise. These results confirm the findings of previous studies, which demonstrated the superior image quality of DLR compared to conventional TSE and IR images for various neuroradiological issues [13,14,29,30,40]. Al Mansour et al. detected superior image noise in T1- and T2-weighted DLR images, but they detected no difference in overall image quality between conventional and DLR TSE sequences [14]. In contrast to our finding of improved image quality, this may be due to the fact that the authors also focused primarily on the assessment of the bony and soft tissue situation, which is different from cerebral imaging. This would also be consistent with the work of Estler et al., who also demonstrated better image quality for DLR images of the lower spine [30], as well as the research of Gassenmaier et al., who also demonstrated better image quality of DLR images for the assessment of the prostate parenchyma compared to conventional TSE sequences [15,39]. In addition, Estler et al. showed the improved overall image quality, sharpness, lesion delineation, and diagnostic confidence, as well as less image noise and artifacts, of DLR FLAIR images compared to traditional FLAIR images in cerebral imaging [13], which is fully consistent with our results in terms of image quality assessment. Previous studies have already addressed the phenomenon that DLR images have even less image noise compared to fully scanned conventional imaging techniques and that this characteristic leads to DL images appearing artificial to experienced radiologists [5,41].

The diagnostic confidence analysis revealed no evidence between the protocols. These results reflect the work of previous studies on diagnostic confidence in various neuroradiologic and abdominopelvic questions, which consistently showed at least equivalent or improved diagnostic confidence using DLR [13,15,29,30,36,37,39,42].

However, this research also highlights other valuable potential applications for DLR, including improving image quality by capturing high-resolution images. Such improvements could increase the image information of T1- and T2-weighted TSE sequences, revealing anatomical details that were previously invisible. In some cases, we noticed that the internal structures of the tumor mass and septations were much clearer in the DLR images, which we also attributed to the higher in-plane resolution of the sequences. Particularly in uncooperative patients, we identified some cases showing significant movement artifacts on conventional TSE sequences that were not diagnostically evaluable. The use of DLR sequences in these patients provided significant benefits, including high diagnostic confidence, tumor conspicuity, and image quality due to the absence of movement artifacts. Furthermore, an additional repeated scan could be avoided.

In the follow-up MRI examinations of glioblastoma patients, the early detection of suspicious lesions and improved delineation of tumorous and therapy-associated signal alterations have the potential to improve patient care. Nevertheless, no significant differences in lesion detection were observed in this study, partly because changes in the final RANO response require not only new lesions but also a relatively major change in the remaining contrast-enhanced or non-contrast-enhanced lesions.

Regarding tumor classification according to RANO 2.0, there was no significant difference in the dimensions and volume of ET, ENT, and NENT lesions. Furthermore, the detection of new lesions and the final RANO statement were not negatively influenced by the DLR sequences. This is consistent with the work of Gassenmaier et al., who also revealed no difference in lesion size measurements between conventional and DLR T2-weighted sequences in abdominopelvic imaging [15,36]. This is important in order not to obtain a different tumor size and thus ultimately a different response assessment. Nevertheless, it should be noted that there is no imaging gold standard for the true extent of tumor size.

This study has some limitations. First, the study included only 33 patients, but this number was determined by power analysis. Nevertheless, the sample size of this study was larger than that of previous studies investigating the usefulness of DL for improving image quality in neuroradiological imaging [13], but further investigations with larger sample sizes are necessary to validate the findings and to explore potential artificial findings or masking of pathologies. Second, we focused on glioblastoma and excluded many patients with other brain tumors, potentially introducing selection bias and impacting the results’ generalizability. Further research is needed to transfer the results to other subgroups of brain tumors. Third, a qualitative imaging study was conducted that was closely focused on clinical diagnostic routine. Consequently, quantitative measurements of SNR and CNR were not employed, as these do not play a role in everyday clinical practice and are also the subject of controversial discussion in the case of MRI images due to the inhomogeneous noise [43]. However, further studies are required to address this issue in a systematic manner using phantom measurements. Fourth, we included various age groups and genders. The heterogeneity of the population might introduce confounding factors that could impact the study’s outcomes. Nevertheless, we chose this prerequisite-free setup in light of the clinical routine of our university hospital. Fifth, the subjective analysis relied on the ratings from two neuroradiologists, and these lesion measurements were performed by one neuroradiologist; the RANO interpretation was based on two neuroradiologists. Although there was a strong inter-rater agreement, subjective assessments can be influenced by individual biases, potentially affecting the reliability of the results. Last, this study used MRI examinations from a single scanner by one vendor and a commercially available product. Therefore, the generalizability of our results beyond this setup may be limited.

## 5. Conclusions

The feasibility of a DLR-optimized glioblastoma protocol was demonstrated by a 30% reduction in acquisition time on average, improved subjective image quality, and the maintenance of high diagnostic accuracy in tumor detection and tumor response assessment according to RANO 2.0, as compared to conventional sequences. In patients with severe glioblastoma, the time window for obtaining high-quality images can be more effectively utilized, leading to a reduction in the number of necessary repeat measurements due to images that are not diagnostically useful, as well as a reduction in the number of aborted examinations.

## Figures and Tables

**Table 1:** MRI acquisition parameters.

Parameters	FLAIR_S_	FLAIR_DLR_	T2_S_	T2_DLR_	T1CE_S_	T1CE_DLR_
Field of view (mm)	230	230	230	230	230	230
Voxel size (mm)	0.7 × 0.7 × 4.0	0.4 × 0.4 × 4.0	0.6 × 0.6 × 4.0	0.3 × 0.3 × 4.0	0.8 × 0.8 × 4.0	0.4 × 0.4 × 4.0
Slice thickness (mm)	4	4	4	4	4	4
Number of slices	40	40	40	40	40	40
Base Resolution	320	320	384	384	304	304
Parallel imaging factor	2	4	2	4	2	4
Acceleration mode	GRAPPA	GRAPPA	GRAPPA	GRAPPA	GRAPPA	GRAPPA
Reference Lines	72	72	72	72	72	72
TR (ms)	8800	8800	4220	4220	2170	2170
TE (ms)	81	81	82	82	9.7	9.7
Averages	1	1	1	1	1	1
Concatenations	2	2	2	2	2	2
Acquisition time (min)	2:40	1:57	1:09	0:51	2:03	1:19

FLAIR = fluid-attenuated inversion recovery; S = standard acquisition technique; DLR = deep learning-reconstructed technique; CE = contrast-enhanced; GRAPPA = Generalized Autocalibrating Partial Parallel Acquisition (parallel imaging technique); TR = repetition time; TE = echo time.

**Table 2:** Patients’ characteristics.

Characteristics	Values
Number of patients	*n* = 33
Age, mean ± standard deviation (years)	59.8 ± 10.6
Sex (male vs. female)	*n* = 19 (57.7%) vs. *n* = 14 (42.4%)
Initial diagnosis without previous therapy	*n* = 2 (6%)
Time of imaging since first diagnosis (mean ± standard deviation (SD, months)	23 ± 27.2
Clinical Scores	
Karnofsky Performance Scale Index (KPS in %), median [interquartile range]	70 [40–100]
ECOG Performance Status Scale, median [interquartile range]	1 [0–2]
Neurologic Assessment in Neuro-Oncology (NANO), median [interquartile range]	2 [0–4]
Montreal Cognitive Assessment (MoCA) *, median [interquartile range]	26 [21.5–27.5]
Mini-Mental State Examination (MMSE) *, median [interquartile range]	28.5 [26–30]
Distress thermometer (DT) *, median [interquartile range]	4.5 [2–5.75]
Therapy	*n* = 31/33
These 31 patients received the following therapy:	
Surgery	*n* = 31/31 (100%)
Radiotherapy	*n* = 31/31 (100%)
Bevacizumab	*n* = 7/31 (23%)
Immunotherapy	*n* = 3/31 (10%)
Temozolomide	*n* = 17/31 (54%)
PCV scheme	*n* = 2/31 (6%)
CeTeG	*n* = 2/31 (6%)
Lomustine	*n* = 6/31 (19%)

* MOCA was determined in 22/33 patients, MMSE in 21/33 patients and DT in 32/33 patients. ECOG = Eastern Cooperativ Oncology Group; CeTeG = lomustine/temozolomide combination therapy; PCV = procarbazine, lomustine, and vincristine

**Table 3:** Time savings using DLR sequences.

Parameters	Standard Acquisition Time in Min	DLR Acquisition Time in Min	Time Saving in Min
FLAIR	2:40	1:57	0:43 (27%)
T2	1:09	0:51	0:18 (26%)
T1CE	2:03	1:19	0:44 (36%)
Time saving (on average)			0:35 (30%)

DLR = deep learning-reconstructed technique; FLAIR = fluid-attenuated inversion recovery; CE = contrast-enhanced.

**Table 4:** Median (interquartile range) image quality in standard (FLAIR_S_) and deep learning-reconstructed fluid-attenuated inversion recovery imaging (FLAIR_DLR_).

Characteristics	Rater 1			Rater 2		
	FLAIR_S_	FLAIR_DLR_	*p*-Value	FLAIR_S_	FLAIR_DLR_	*p*-Value
Image noise	4 [4–4]	5 [5–5]	<0.001	4 [4–4]	5 [5–5]	<0.001
Artifacts	5 [5–5]	5 [5–5]	0.180	5 [5–5]	5 [5–5]	0.083
Sharpness	4 [3–4]	5 [5–5]	<0.001	3 [3–4]	5 [4–5]	<0.001
Overall image quality	4 [4–4]	5 [5–5]	<0.001	4 [4–4]	5 [5–5]	<0.001
Tumor conspicuity	4 [4–4]	5 [5–5]	<0.001	4 [4–4]	5 [5–5]	<0.001
Diagnostic confidence	5 [5–5]	5 [5–5]	0.317	5 [5–5]	5 [5–5]	0.317

S = standard acquisition technique; DLR = deep learning-reconstructed technique; Likert-scale ranging from 1–5, with 5 being the best rating.

**Table 5:** Median (interquartile range) image quality in standard T2-weighted (T2_S_) and deep learning-reconstructed T2-weighted imaging (T2_DLR_).

Characteristics	Rater 1			Rater 2		
	T2_S_	T2_DLR_	*p*-Value	T2_S_	T2_DLR_	*p*-Value
Image noise	4 [4–4]	5 [5–5]	<0.001	4 [4–4]	5 [5–5]	<0.001
Artifacts	5 [5–5]	5 [4–5]	0.132	5 [4.5–5]	5 [4–5]	0.180
Sharpness	4 [4–4]	5 [5–5]	<0.001	4 [4–4]	5 [5–5]	<0.001
Overall image quality	4 [4–4]	5 [5–5]	<0.001	4 [4–4]	5 [5–5]	<0.001
Tumor conspicuity	4 [4–4]	5 [5–5]	<0.001	4 [4–4]	5 [5–5]	<0.001
Diagnostic confidence	5 [5–5]	5 [5–5]	0.157	5 [5–5]	5 [5–5]	0.083

S = standard acquisition technique; DLR = deep learning-reconstructed technique; Likert-scale ranging from 1–5, with 5 being the best rating.

**Table 6:** Median (interquartile range) image quality in standard contrast-enhanced T1-weighted (T1CE_S_) and deep learning-reconstructed contrast-enhanced T1-weighted imaging (T1CE_DLR_).

Characteristics	Rater 1			Rater 2		
	T1CE_S_	T1CE_DLR_	*p*-Value	T1CE_S_	T1CE_DLR_	*p*-Value
Image noise	4 [4–4]	5 [5–5]	<0.001	4 [3–4]	5 [5–5]	<0.001
Artifacts	4 [4–4]	4 [4–4]	0.046	4 [4–4]	4 [4–4]	0.157
Sharpness	3 [3–4]	5 [5–5]	<0.001	3 [3–4]	5 [5–5]	<0.001
Overall image quality	4 [4–4]	5 [5–5]	<0.001	4 [3–4]	5 [5–5]	<0.001
Tumor conspicuity	4 [4–4]	5 [5–5]	<0.001	4 [4–4]	5 [5–5]	<0.001
Diagnostic confidence	5 [4–5]	5 [4.5–5]	0.414	5 [4–5]	5 [4.5–5]	0.317

CE = contrast-enhanced; S = standard acquisition technique; DLR = deep learning-reconstructed technique; Likert-scale ranging from 1–5, with 5 being the best rating.

**Table 7:** Mean (range) interrater intraprotocol agreement between rater 1 and rater 2 using Cohen’s kappa and Kendall’s Tau.

	Cohen’s Kappa	Kendall’s Tau
Overall image quality_S_	0.672 (0.577–0.787)	0.696 (0.571–0.821)
Overall image quality_DLR_	0.632 (0.476–0.784)	0.695 (0.559–0.803)
Diagnostic confidence_S_	0.701 (0.570–0.748)	0.747 (0.570–0.784)
Diagnostic confidence_DLR_	0.632 (0.459–0.784)	0.655 (0.459–0.803)

DLR = deep learning-reconstructed technique; S = standard acquisition technique.

## Data Availability

In order to safeguard the confidentiality of the participants, the data pertaining to this study are currently withheld from public access. The data can be shared upon request.
